# Factors Influencing Decision-Making in Companion Animal Euthanasia: A Mixed-Methods Study of Pet Owners and Veterinarians

**DOI:** 10.3390/ani16111738

**Published:** 2026-06-05

**Authors:** Annamária Kiss, Wieka Möller, Zsombor Wagenhoffer, Kinga Fodor

**Affiliations:** 1 Department of Laboratory Animal Science and Animal Welfare, University of Veterinary Medicine Budapest, 1078 Budapest, Hungary; wiekamoeller@icloud.com (W.M.); fodor.kinga@univet.hu (K.F.); 2 Institute for Animal Breeding, Nutrition and Laboratory Animal Sciences, University of Veterinary Medicine Budapest, 1078 Budapest, Hungary; wagenhoffer.zsombor@univet.hu

**Keywords:** animal welfare, euthanasia, companion animal, veterinary communication, quality of life, decision-making, end-of-life care

## Abstract

Euthanasia in companion animal practice represents one of the most emotionally and ethically challenging decisions in veterinary medicine, requiring clinical judgment, effective communication, and sensitivity toward both animal welfare and pet owner well-being. This mixed-methods exploratory study investigated decision-making in small animal euthanasia from both pet owner and veterinarian perspectives. An online questionnaire completed by 228 pet owners from 17 countries was supplemented by two semi-structured interviews involving small animal veterinarians with different professional backgrounds. The qualitative interview component was exploratory and was used to contextualize the survey findings. Quantitative data were analyzed using descriptive and inferential statistics, while qualitative responses were examined thematically. Illness (63.3%) and age-related decline (31.6%) were the most frequently reported reasons for euthanasia. Most pet owners assessed their animal’s condition through personal observation (53.5%) or veterinary advice (39.2%), whereas structured quality-of-life tools were rarely used (7.3%). Emotional attachment represented the most influential factor in decision-making (69.3%). Pet owners who explicitly reported receiving emotional support from their veterinarian experienced significantly lower emotional burden (Holm-adjusted *p* = 0.002) and greater satisfaction with communication (Holm-adjusted *p* = 0.006) than those who explicitly reported no emotional support. Interview findings emphasized medical justification, individualized communication, and ethical responsibility. These findings highlight the central role of communication, emotional support, and structured end-of-life guidance in improving companion animal euthanasia decision-making. Because the study relied on voluntary online recruitment and included a limited qualitative sample, the findings should be interpreted as exploratory.

## Simple Summary

Deciding when it is the right time to euthanize a companion animal is one of the most difficult situations faced by both pet owners and veterinarians. This decision is not based only on medical facts, but is also strongly influenced by emotions, ethical concerns, personal beliefs, and the relationship between people and their animals. The aim of the study was to better understand how pet owners and veterinarians experience and manage this decision, with a special focus on communication, emotional burden and the assessment of an animal’s quality of life. To explore these questions, an international survey involving 228 pet owners from 17 countries was conducted, supplemented by two exploratory semi-structured interviews with small animal veterinarians from different professional backgrounds. The results showed that pet owners often rely on their personal observations and emotional connection with their pet rather than using structured quality-of-life tools. Emotional attachment was the strongest factor influencing decision-making. Pet owners who felt supported by their veterinarian reported less emotional distress and greater satisfaction with the euthanasia process. These findings highlight the importance of compassionate communication, emotional support, and better training in end-of-life care, helping veterinarians support both animal welfare and the well-being of pet owners.

## 1. Introduction

Euthanasia is one of the most ethically complex and emotionally demanding responsibilities in companion animal practice. Its primary purpose is to prevent unnecessary suffering and provide a humane death when recovery is no longer possible [1]. However, the decision extends far beyond a clinical procedure and often represents a profound emotional event for both veterinarians and pet owners [2,3,4]. Determining the appropriate timing of euthanasia requires the integration of medical evidence, quality-of-life assessment, ethical reasoning, and sensitive communication, making it one of the most challenging aspects of veterinary decision-making [5,6]. In companion animal medicine, euthanasia decisions are further complicated by the strong emotional bond between pet owners and their animals, which may transform clinical end-of-life choices into deeply personal and ethical dilemmas. Both premature and unnecessarily delayed euthanasia may therefore raise important welfare concerns, requiring veterinarians to balance medical indicators with the emotional significance of the human–animal relationship [7]. Contemporary veterinary ethics increasingly describe euthanasia not merely as the prevention of suffering, but as the facilitation of a “good death”, in which clinical judgment, animal welfare, ethical justification, and pet owner-related circumstances must be carefully balanced. This broader perspective emphasizes that end-of-life decision-making requires not only technical competence, but also moral reflection and individualized case-based evaluation [8]. Advances in veterinary medicine have increasingly blurred the boundary between prolonging life and preserving quality of life, raising important ethical questions regarding whether euthanasia should be considered solely in response to pain and suffering, or also in situations where positive welfare and meaningful quality of life can no longer be maintained [9]. Ethical decision-making may be further complicated by differences in legal frameworks, cultural expectations, and individual moral values across clinical and societal contexts [10].

In recent years, increasing attention has been directed toward the emotional and psychological consequences of euthanasia for veterinary professionals and animal caregivers. Studies have shown that veterinarians frequently experience moral distress when euthanasia is requested prematurely in animals with treatable conditions or, conversely, when pet owners insist on continued treatment despite obvious suffering and poor welfare prospects [11,12,13,14]. Repeated exposure to ethically challenging end-of-life decisions has also been associated with emotional exhaustion, compassion fatigue, and burnout within the veterinary profession [15,16]. In the context of this study, emotional burden refers to the subjective psychological and emotional strain experienced during euthanasia-related decision-making. This may include grief, guilt, uncertainty, decisional conflict, anticipatory loss, and distress associated with balancing the animal’s welfare against the pet owner’s emotional attachment. In veterinary professionals, emotional burden may also include moral distress, compassion fatigue, and the repeated psychological impact of participating in end-of-life decisions. In a Hungarian questionnaire study involving 211 veterinarians, 67.8% of respondents reported that some euthanasia cases affected them emotionally to varying degrees, and 63.5% stated that they had cried during or after performing euthanasia [17]. Beyond the prevention of pain and suffering, contemporary animal welfare concepts increasingly emphasize whether an animal continues to experience “a life worth living”, integrating both physical condition and affective well-being into end-of-life decision-making [18]. This quality-of-life-oriented perspective may provide veterinarians and pet owners with a more ethically grounded framework when evaluating whether euthanasia is in the animal’s best interest. Veterinarians frequently act as professional “gatekeepers” at the end of an animal’s life, balancing the interests of the patient, the pet owner, and the clinical circumstances during euthanasia-related decision-making. Ethically sound end-of-life decisions are therefore increasingly supported by structured communication, quality-of-life assessment tools, and clearly defined clinical protocols [19]. Similarly, pet owners often report intense grief, guilt, uncertainty, and decisional conflict when confronted with end-of-life choices for a companion animal [20,21]. The grieving process associated with companion animal loss may begin before euthanasia is performed, as pet owners often experience anticipatory grief while facing progressive illness and end-of-life decisions. Studies have shown that adequate preparation, emotional validation, and active involvement in decision-making may reduce guilt, decisional uncertainty, and maladaptive grief responses following the loss of a companion animal [22]. The experience of companion animal euthanasia is also strongly influenced by the quality of communication between veterinarians and pet owners. Previous studies have shown that clear information, empathetic communication, and emotional support provided by the veterinary team may improve pet owners’ confidence in end-of-life decisions, reduce uncertainty, and contribute to a more positive coping process following the loss of a companion animal [23]. Nursing-focused literature has further highlighted that pet owner experiences during companion animal euthanasia may be significantly improved by clear procedural guidance, adequate time for farewell, emotional reassurance, privacy, and continued bereavement support before, during, and after the procedure [24].

To support clinical decision-making, several structured quality-of-life assessment tools have been developed for companion animals, including multidimensional scoring systems designed to evaluate pain, mobility, appetite, hygiene, and overall well-being [5,25,26]. Although these tools may improve objectivity and facilitate shared decision-making, previous research suggests that their implementation in routine clinical practice remains inconsistent, with many pet owners relying primarily on personal observation, emotional attachment, or informal veterinary advice instead of standardized assessments [25,27,28]. Ethical frameworks for veterinary euthanasia further suggest that decision-making should not rely solely on the animal’s current condition, but also on its expected welfare trajectory until the next realistic clinical reassessment. This prospective approach reinforces the importance of repeated quality-of-life evaluation and shared decision-making throughout progressive disease management [29]. In practical end-of-life contexts, quality-of-life assessment tools generally evaluate several welfare-related domains, such as pain, mobility, appetite, hydration, hygiene, respiratory comfort, social interaction, interest in daily activities, and the balance between good and bad days [5,25,26]. Examples include multidimensional tools such as the HHHHHMM scale, which considers hurt, hunger, hydration, hygiene, happiness, mobility, and whether there are more good days than bad days, as well as broader welfare-based approaches that integrate physical health, behavior, and affective state [25,26,28]. These tools are intended to support structured reflection and shared decision-making. Their value lies in making complex and emotionally charged decisions more explicit, repeatable, and communicable between veterinarians and pet owners [25,27,28].

Communication between veterinarians and pet owners has repeatedly been identified as a critical determinant of end-of-life experiences. Compassionate communication, clear explanation of prognosis, and emotional support have been associated with improved pet owner satisfaction, reduced decisional regret, and better acceptance of euthanasia [26,29]. However, cultural differences, financial limitations, personal beliefs, and conflicting perceptions of animal suffering may complicate communication and create ethical tension between professional recommendations and pet owner expectations [3,11,28]. These challenges highlight the veterinarian’s dual role not only as a medical expert, but also as an ethical advisor and emotional mediator. End-of-life decision-making in veterinary medicine also carries important ethical and legal dimensions, as euthanasia requires balancing animal welfare, pet owner autonomy, professional responsibility, and societal expectations. In cases involving uncertainty, disagreement or conflicting interests, veterinarians may therefore face not only clinical challenges, but also moral and legal accountability regarding whether euthanasia truly serves the best interests of the animal [29,30].

Despite growing interest in veterinary ethics and end-of-life care, relatively little empirical research has examined euthanasia decision-making from both pet owner and veterinarian perspectives within the same study, particularly using mixed-methods approaches that combine quantitative and qualitative data [6,20]. Furthermore, the role of emotional support, communication quality, and quality-of-life assessment in shaping euthanasia experiences remains insufficiently understood in an international context.

Therefore, the aim of the study was to investigate the factors influencing decision-making in companion animal euthanasia from the perspectives of pet owners and veterinarians. Using an international survey combined with semi-structured interviews, this study explored the clinical, emotional, ethical, and communicative factors involved in determining the appropriate timing of euthanasia. The findings suggest emotional attachment, veterinary communication, and perceived professional support play central roles in pet owner experiences, emphasizing the importance of structured end-of-life guidance and communication training in veterinary practice.

## 2. Materials and Methods

### 2.1. Study Design

**Figure 1:**
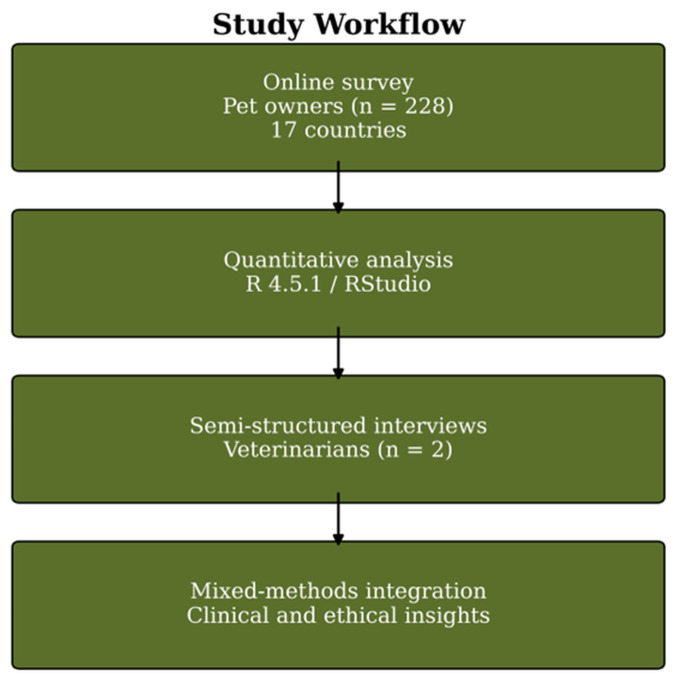
Study workflow and mixed-methods structure of the exploratory investigation of companion animal euthanasia decision-making. Quantitative data were collected through an international online survey involving pet owners and caregivers (*n* = 228) from 17 countries. The survey data were analyzed using descriptive statistics and exploratory inferential tests in R software. The qualitative component consisted of two semi-structured veterinarian interviews, which were used to provide professional context and illustrative insight. Quantitative and qualitative findings were integrated during interpretation to identify clinical, ethical, emotional, and communication-related aspects of euthanasia decision-making.

The study employed an exploratory mixed-methods design combining quantitative survey data with qualitative semi-structured interviews to investigate factors influencing decision-making in companion animal euthanasia from both pet owner and veterinarian perspectives. The mixed-methods approach was selected to provide both statistical insight into pet owner experiences and in-depth understanding of professional ethical and emotional perspectives among veterinarians. The study was exploratory and descriptive in nature and was designed to identify patterns, associations, and recurring themes rather than to test causal relationships. The overall study workflow is presented in Figure 1.

### 2.2. Survey Study

#### 2.2.1. Survey Design and Data Collection

A cross-sectional online questionnaire was developed to investigate pet owners’ experiences with companion animal euthanasia, including decision-making processes, emotional burden, quality-of-life assessment, and satisfaction with veterinary communication. Data collection was conducted between 13 January 2025 and 1 April 2025. The survey was distributed through social media platforms using a publicly accessible URL link. The questionnaire was created using Microsoft Forms and was available in both English and German. English was selected to allow broader international participation, while German was included because a substantial part of the authors’ recruitment network and expected respondent population was located in German-speaking contexts. Although the survey URL was publicly accessible and could be shared beyond the authors’ immediate networks. Additional language versions were not prepared because validated translations were not available at the time of data collection and because the study was designed as an exploratory survey. The authors acknowledge that limiting the questionnaire to English and German may have reduced participation from non-English- and non-German-speaking pet owners and may have contributed to the predominance of German respondents in the final sample.

The questionnaire was a study-specific, purpose-designed instrument developed for the aims of the present exploratory study. The topics were selected based on the main research objectives, the literature on companion animal euthanasia and end-of-life decision-making, and clinically relevant aspects of owner–veterinarian communication. The questionnaire domains were chosen to capture the key dimensions identified as relevant to euthanasia decision-making: demographic characteristics, previous involvement in euthanasia decisions, reported reasons for euthanasia, quality-of-life assessment, factors influencing the decision, emotional burden, satisfaction with veterinary communication, provision of information before the procedure, perceived emotional support from the veterinarian, and open-ended feedback on the euthanasia experience. It was intended as an exploratory survey tool to describe patterns and associations in pet owners’ experiences. The questionnaire consisted of single-choice, multiple-choice, Likert-type scales, and open-ended questions. Emotional burden and satisfaction with veterinary communication were assessed using five-point Likert-type scales. For emotional burden, higher scores indicated greater burden; for communication satisfaction, lower scores indicated greater satisfaction.

#### 2.2.2. Participants and Inclusion Criteria

Eligible participants were pet owners or caregivers aged 18 years or older who had either made or actively participated in a euthanasia decision involving a companion animal. The survey was distributed through a publicly accessible URL on social media platforms. The total number of individuals who viewed the invitation, opened the questionnaire, or started but did not submit it could not be determined. Microsoft Forms exported only fully submitted questionnaires for analysis. Therefore, partial or abandoned responses were not available in the final dataset, and the overall completion rate could not be reliably assessed. A total of 228 complete responses from participants representing 17 countries were therefore included in the analysis. The URL link, which allowed the study to reach pet owners and caregivers who had previous personal experience with companion animal euthanasia. This sampling approach was selected because the target population was specific, emotionally sensitive, and geographically dispersed, and because the exploratory aim of the study was to collect owner experiences rather than to obtain a nationally representative sample. The animal species involved in the euthanasia decision was not recorded. This decision was made because the questionnaire primarily focused on the pet owner’s decision-making experience, emotional burden, communication with the veterinarian, and use of quality-of-life assessment. Eligibility was based on self-report. For consistency, euthanasia was defined at the beginning of the questionnaire as the intentional termination of life to prevent pain and suffering.

### 2.3. Qualitative Interviews

The qualitative component was designed as a small exploratory interview strand intended to provide professional context for the survey findings. Only two veterinarians were interviewed because the primary focus of the study was the quantitative survey of pet owners. The interviews were therefore used in a complementary and illustrative role. The interviewed veterinarians were practicing in Germany partly because most completed questionnaires came from Germany, making this clinical context particularly relevant to the survey sample. However, they were selected primarily because they represented contrasting professional backgrounds and markedly different levels of clinical experience. One participant was an experienced small animal practitioner in Germany with more than 36 years of professional experience, while the second participant was an early-career veterinarian originally from Brazil and currently practicing in Germany with approximately two years of clinical experience.

Interviews were conducted individually, recorded with participant consent, and subsequently transcribed for thematic analysis. A semi-structured interview guide was used to ensure that comparable topics were addressed in both interviews while allowing participants to elaborate on their individual experiences. The guide included questions on euthanasia frequency, decision-making criteria, quality-of-life assessment, communication with pet owners, ethical dilemmas, emotional burden, professional training, and perceived educational needs. Interview-derived information was anonymized during analysis, and only professionally relevant details were retained in the manuscript.

### 2.4. Data Analysis

#### 2.4.1. Quantitative Analysis

Survey responses were exported from Microsoft Forms into Microsoft Excel for data cleaning and coding. Responses were checked for completeness, consistency, and obvious entry errors. Multiple-response questions were separated into individual binary variables before analysis. Open-ended responses were reviewed and categorized where appropriate for descriptive analysis, while longer narrative responses were retained for qualitative thematic interpretation. Percentages were calculated using the total number of respondents included in the final analysis unless otherwise stated.

Statistical analyses were performed using R software (version 4.5.1; R Core Team, 2025). Descriptive statistics were calculated for demographic variables and questionnaire responses. Categorical variables were summarized as frequencies and percentages. Likert-type scale variables, including emotional burden and satisfaction with veterinary communication, were analyzed using both descriptive and exploratory inferential approaches. Because the response categories were ordered and the five-point Likert-type scales were symmetrically structured, mean values, standard deviations, medians, and full response distributions were reported to provide a transparent summary of the data.

Welch’s two-sample *t*-tests were used for exploratory comparisons of mean scores between two groups, including respondents who did or did not report receiving emotional support from the veterinarian and those who were or were not informed about the euthanasia procedure in advance. For group comparisons involving perceived emotional support from the veterinarian, only explicit “Yes” and “No” responses were included. Ambiguous free-text responses, “not applicable” answers, or responses that did not clearly indicate perceived support were excluded from this specific comparison. One-way analysis of variance was used to compare emotional burden across age groups.

Effect sizes were calculated to complement *p*-values. Cohen’s d was used for two-group comparisons, and eta-squared was used for the one-way analysis of variance. For the three planned owner-experience comparisons *p*-values were additionally adjusted using the Holm–Bonferroni method to reduce the risk of type I error due to multiple testing. Results based on Likert-type data were interpreted cautiously and together with descriptive response distributions. Statistical significance was set at *p* < 0.05.

#### 2.4.2. Qualitative Analysis

Interview transcripts and open-ended survey responses were analyzed using descriptive thematic analysis. The analysis focused on identifying recurring patterns related to ethical responsibility, communication, emotional burden, cultural and financial influences, and professional decision-making. Themes were developed iteratively through repeated reading of the responses and comparison across survey feedback and interview material. The qualitative findings were used to contextualize and interpret the quantitative survey results.

### 2.5. Ethical Considerations

Participation in both the survey and interviews was voluntary. Survey participants were informed about the purpose of the study, the anonymous nature of data collection, the voluntary nature of participation, and their right to withdraw before submitting the questionnaire. No directly identifying personal data were collected from survey respondents. Completion and submission of the anonymous online questionnaire were considered informed consent to participate.

The veterinarian interview participants were informed about the purpose of the interview, voluntary participation, audio recording, transcription, anonymized analysis, and the use of interview-derived information for scientific publication. Verbal informed consent was obtained before each interview and before audio recording. Interview-derived information was anonymized during analysis, and only professionally relevant information was retained in the manuscript.

According to applicable institutional and national regulations, formal ethical approval was not required for this anonymous, non-interventional survey-based study involving adult participants. The study did not involve experimental intervention, identifiable human medical data, or identifiable patient-level animal clinical records.

### 2.6. Generative Artificial Intelligence Use Statement

No generative artificial intelligence tools were used in the collection, analysis, or interpretation of data in this study.

## 3. Results

### 3.1. Survey Population

A total of 228 completed questionnaires were included in the final analysis. Respondent characteristics are summarized in Table 1. After harmonization of country names, respondents represented 17 countries. The largest groups were from Germany (*n* = 157, 68.9%), Hungary (*n* = 27, 11.8%), and South Africa (*n* = 15, 6.6%). The remaining respondents came from Austria, France, Ireland, India, Switzerland, the United Arab Emirates, Israel, Malta, the Netherlands, Norway, South Korea, Spain, the United Kingdom, and the United States. Most respondents were young adults, and the sample was predominantly female. All participants had previously experienced or actively participated in the euthanasia decision of a companion animal.

### 3.2. Reported Reasons for Companion Animal Euthanasia

**Figure 2:**
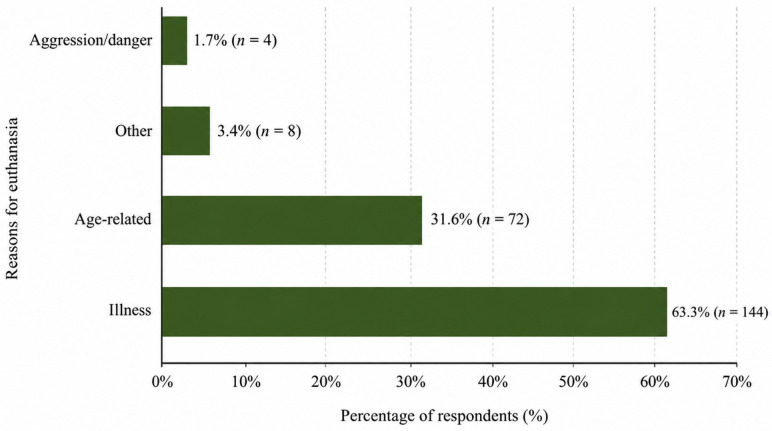
Reported reasons for euthanasia of companion animals (*n* = 228). Percentages and absolute frequencies are shown for each response category. Multiple responses were allowed; therefore, percentages do not necessarily sum to 100%.

Illness was reported as the most common reason for euthanasia (63.3%), followed by age-related decline (31.6%). Less frequently reported reasons included behavioral aggression (1.7%) and other causes such as trauma, blindness, or neglect (3.4%) (Figure 2). These findings indicate that medical and age-associated conditions represented the primary clinical indications for euthanasia among surveyed pet owners.

### 3.3. Quality of Life Assessment Prior to Euthanasia

**Figure 3:**
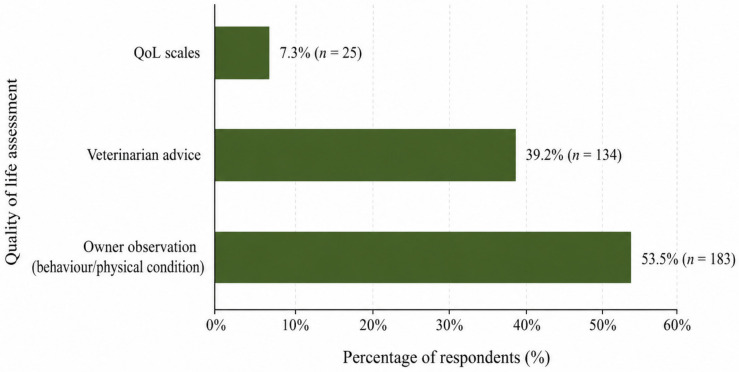
Methods used by respondents to assess their pet’s quality of life prior to euthanasia (*n* = 228). Percentages and absolute frequencies are displayed for each category. Multiple responses were allowed; therefore, percentages do not necessarily sum to 100%.

When evaluating their animal’s condition before euthanasia, most respondents relied on personal observation of behavior, physical condition, and daily functioning (53.5%), including observations such as changes in appetite, mobility, activity level, social interaction, visible signs of discomfort or pain, respiratory comfort, hygiene, and the animal’s ability to perform normal daily routines. Veterinary advice was identified as an important source of guidance by 39.2% of participants. In contrast, structured quality-of-life assessment tools were used by only 7.3% of respondents, indicating limited implementation of standardized assessment methods in routine decision-making (Figure 3).

### 3.4. Factors Influencing Decision-Making

**Figure 4:**
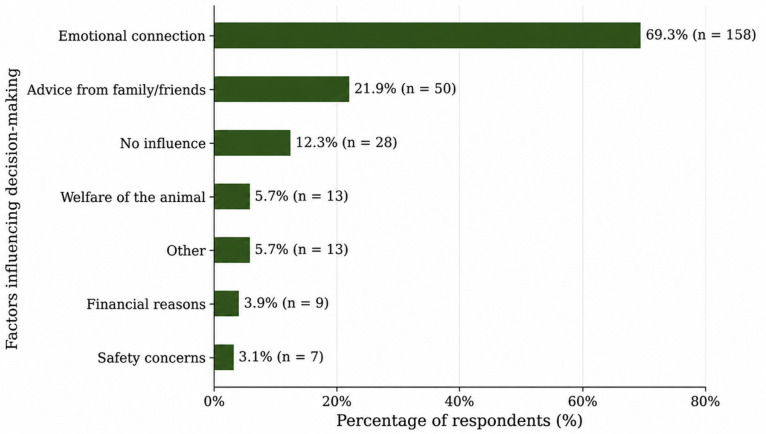
Factors influencing the decision for companion animal euthanasia (*n* = 228). Percentages and absolute frequencies are presented for each category. Multiple responses were allowed; therefore, percentages do not necessarily sum to 100%.

Emotional attachment to the animal was the most frequently reported factor influencing euthanasia decisions (69.3%). Advice from family members or friends was reported by 21.9% of respondents, whereas financial considerations (3.9%), animal welfare concerns (5.7%), and safety-related reasons (3.1%) were reported less frequently. A minority of respondents (12.3%) indicated that no factors influenced their decision (Figure 4).

### 3.5. Emotional Burden and Veterinary Communication

The euthanasia decision was perceived as emotionally challenging, with a mean emotional burden score of 3.43 ± 1.34 on a five-point Likert-type scale. The median score of 4 indicated that most respondents experienced moderate to high emotional distress during the decision-making process. Neither age (one-way analysis of variance: *F*(5, 222) = 1.17, *p* = 0.325) nor gender (*t* = 0.50, *p* = 0.619) showed a statistically significant association with emotional burden.

Overall satisfaction with veterinary communication during the euthanasia process was high, with a mean score of 1.96 ± 1.36 on the five-point Likert-type scale, where lower values represented greater satisfaction. However, responses showed substantial variability between participants.

**Figure 5:**
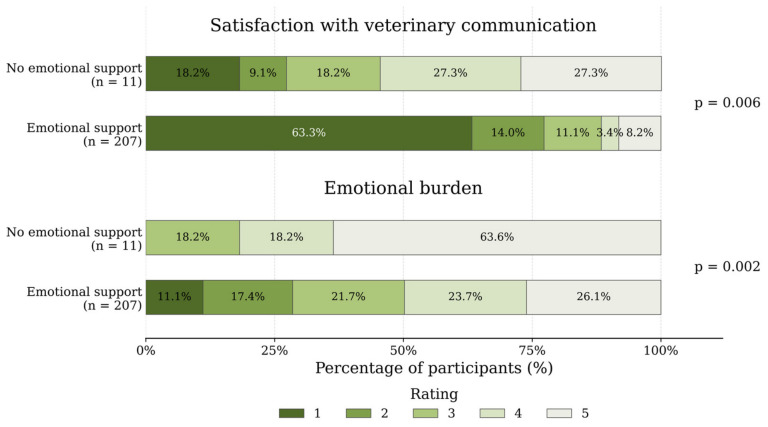
Distribution of satisfaction with veterinary communication and emotional burden according to perceived emotional support from the veterinarian. Only respondents who explicitly answered “Yes” or “No” to the emotional support question were included in this comparison. Responses were rated on five-point Likert-type scales. For communication satisfaction, lower scores indicate greater satisfaction, whereas for emotional burden, higher scores indicate greater burden. Percentages are shown within response categories. Holm-adjusted *p*-values are displayed.

More than 90% of respondents (90.4%) reported receiving information prior to the euthanasia procedure. Pet owners who explicitly reported receiving emotional support from their veterinarian experienced significantly lower emotional burden than those who explicitly reported not receiving support (3.36 ± 1.33 vs. 4.45 ± 0.82; *t* = −4.14; Cohen’s d = −0.83; Holm-adjusted *p* = 0.002). They also reported significantly greater satisfaction with veterinary communication (1.79 ± 1.26 vs. 3.36 ± 1.50; *t* = −3.41; Cohen’s d = −1.24; Holm-adjusted *p* = 0.006). In addition, respondents who were informed about the euthanasia procedure in advance reported greater satisfaction with veterinary communication than those who were not informed (1.81 ± 1.29 vs. 3.32 ± 1.32; *t* = −5.09, Cohen’s d = −1.17; Holm-adjusted *p* < 0.001) (Figure 5).

The main exploratory comparisons related to pet owner emotional burden and satisfaction with veterinary communication are summarized in Table 2, including test statistics, effect sizes, raw *p*-values, and Holm–Bonferroni-adjusted *p*-values. Pet owners who reported receiving emotional support from their veterinarian had significantly lower emotional burden and significantly greater satisfaction with communication. Respondents who were informed about the euthanasia procedure in advance also reported greater satisfaction with veterinary communication.

### 3.6. Qualitative Feedback from Pet Owners

Open-ended feedback was obtained from 106 respondents. Responses were coded thematically, and a single response could be assigned to more than one theme; therefore, thematic frequencies are not mutually exclusive. The most frequently identified theme was the importance of quality-of-life and animal welfare considerations in euthanasia decision-making (*n* = 31, 29.2%). Respondents emphasized that suffering, loss of quality of life, and the animal’s best interest should remain central to the decision. The second most frequent theme concerned the need for clearer information, explanation, and transparency from veterinarians before euthanasia (*n* = 28, 26.4%). Examples included requests for more detailed explanation of the procedure, clearer discussion of prognosis, and better explanation of how euthanasia drugs act. A further 27 responses (25.5%) did not contain a specific suggestion for improvement or indicated that the respondent was satisfied with the care received.

Empathy, emotional support, and compassionate communication were mentioned in 14 responses (13.2%). Respondents described the importance of gentle communication, emotional sensitivity, and feeling that the veterinarian cared about both the animal and the owner. Calm, private, and comfortable surroundings, as well as sufficient time for farewell, were mentioned in 12 responses (11.3%). Home euthanasia or euthanasia in a familiar environment was suggested in 9 responses (8.5%), while post-euthanasia care, bereavement support, memorial gestures, or support for children were mentioned in 5 responses (4.7%). Illustrative examples are summarized in Table 3.

### 3.7. Veterinarian Interviews

Two veterinarians were interviewed. Because of the limited number of interview participants, the qualitative findings are presented as illustrative professional perspectives. The interviews were used to contextualize the survey findings and to provide insight into how euthanasia-related decision-making may be experienced in clinical practice. Both interviewed veterinarians emphasized that euthanasia decisions should primarily be guided by animal welfare and medical justification. However, differences emerged in how ethical complexity was approached. The main interview findings were summarized in Table 4. This table highlights areas of convergence and divergence between the two veterinarians, particularly regarding medical justification, communication, emotional burden, quality-of-life assessment, and educational preparation.

The senior German veterinarian described strict medical necessity as the primary criterion for euthanasia and reported declining requests considered ethically unjustified. In selected cases, he reported assuming pet ownership of animals to prevent euthanasia that he considered inappropriate.

In contrast, the early-career Brazilian veterinarian working in Germany described a broader decision-making framework that additionally considered the pet owner’s emotional, social, and practical circumstances.

Despite these differences, both veterinarians identified early, individualized, and transparent communication as essential for facilitating informed pet owner decision-making and reducing emotional distress during end-of-life care. Both veterinarians reported that euthanasia could create emotional burden for the veterinarian, although the intensity and specific triggers differed between them. Emotional burden was described as more pronounced in long-term patients, tragic or unexpected cases, young animals, cases involving children, repeated losses within the same family, disagreements with pet owners, uncertainty regarding timing, or situations in which euthanasia was requested either too early or too late from an animal welfare perspective. The veterinarians emphasized the importance of assessing pain, mobility, appetite, behavior, and overall welfare, although formal standardized quality-of-life scales were not described as uniformly integrated into routine decision-making. Both veterinarians were aware of the concept of quality-of-life assessment, but neither described standardized quality-of-life scales as a routinely integrated decision-making tool in daily practice. The senior veterinarian primarily emphasized medical expertise and clinical judgment, whereas the early-career veterinarian referred to clinical judgment, personal experience, and input from the pet owner. This finding is consistent with the survey result showing limited pet owner-reported use of structured quality-of-life tools. To clarify the interview categories, Table 4 provides short illustrative examples of the medical, contextual, emotional, communicative, ethical, and educational aspects mentioned by the two veterinarians.

## 4. Discussion

The study investigated factors influencing decision-making in companion animal euthanasia from both pet owner and veterinarian perspectives using a mixed-methods approach. The findings suggest that euthanasia decision-making is not solely determined by clinical indicators, but is shaped by a complex interaction of emotional attachment, ethical reasoning, communication quality, and perceived professional support. These findings support the initial hypothesis that end-of-life decision-making in veterinary medicine extends beyond medical assessment and represents a multidimensional process involving both clinical and psychosocial factors. The interpretation of these findings is consistent with previous literature showing that euthanasia decision-making in companion animal practice is rarely based on clinical indicators alone [3,5,6,11]. Earlier studies have emphasized that end-of-life decisions are shaped by the animal’s welfare status, the owner–animal bond, communication with the veterinarian, ethical responsibility, and the emotional consequences of loss [20,21,22,23,26,29]. The present findings extend this literature by showing, in an international exploratory sample of pet owners, that emotional attachment, perceived veterinary support, and communication quality were central elements of the euthanasia experience.

One of the most notable findings of the study was that emotional attachment represented the most influential factor affecting euthanasia decisions, being reported by nearly 70% of respondents. This finding is consistent with previous studies describing companion animals as family members and emphasizing the strong emotional interdependence between pet owners and their pets [21,31]. Such attachment may strengthen pet owner advocacy for the animal’s welfare, but may also complicate objective assessment of suffering and contribute to delayed euthanasia decisions. This tension reflects one of the central ethical challenges in companion animal practice, where prolonging life may not always coincide with maintaining welfare [3,11].

A second important finding was the limited use of structured quality-of-life assessment tools. Although validated multidimensional instruments for companion animals are increasingly available, only a small proportion of respondents reported using such tools, while most relied on personal observation or veterinary advice [5,25]. This finding supports previous reports suggesting that quality-of-life tools remain underutilized in clinical practice despite their potential to support shared decision-making [6]. Several possible explanations may be considered for the limited use of structured quality-of-life assessment tools. First, many pet owners may be unaware that such tools exist, particularly if they are not introduced proactively during veterinary consultations [5,25,27]. Second, euthanasia decisions are often made in emotionally charged and time-sensitive circumstances, in which owners may rely more strongly on personal observation, intuition, and perceived suffering than on formal scoring systems [20,21,22,23]. Third, some owners may find numerical or checklist-based tools difficult to apply to a deeply personal relationship with an individual animal, especially when emotional attachment, guilt, and anticipatory grief are present [20,21,22]. Fourth, veterinarians may not routinely integrate formal quality-of-life scales into end-of-life discussions because of time constraints, limited familiarity with specific tools, concern that scoring systems may appear impersonal, or a preference for individualized clinical judgment [5,25,27,28].

The limited use of such tools does not necessarily indicate a lack of concern for animal welfare. Rather, it may reflect a gap between the availability of structured assessment instruments and their practical implementation in emotionally complex owner–veterinarian interactions. From a clinical perspective, quality-of-life tools may be most useful when introduced before the terminal stage, for example during chronic disease management, when owners still have time to observe trends in appetite, mobility, pain, behavior, hygiene, and the balance between good and bad days [25,26,27,28,29]. Presenting these tools as supportive aids rather than rigid decision-making instruments may help owners understand their purpose and may facilitate shared decision-making [5,25,27]. These findings suggest that veterinarians could play an important role in increasing awareness of quality-of-life assessment tools, explaining their limitations, and integrating them sensitively into end-of-life communication.

The results further highlight the central importance of veterinary communication. Pet owners who perceived emotional support from their veterinarian reported significantly lower emotional burden and greater satisfaction with communication. These findings are consistent with the communication framework proposed by Shaw (2006) and with more recent studies highlighting communication as one of the strongest predictors of pet owner satisfaction during end-of-life care [6,32]. This supports earlier work suggesting that communication during end-of-life care should include not only medical explanation, but also emotional validation, preparation for the procedure, and support for owner decision-making. In the present study, this was reflected quantitatively by the association between perceived emotional support and lower emotional burden, and qualitatively by owners’ requests for clearer procedural explanations, empathy, privacy, sufficient time for farewell, and additional emotional support before and after euthanasia. Together, these findings reinforce the view that communication during euthanasia should not be considered an additional professional skill, but rather an essential clinical competency.

The qualitative component represents an important limitation of the study. Only two veterinarians were interviewed, which is insufficient to achieve thematic saturation or to support robust mixed-methods conclusions regarding the wider veterinary profession. Therefore, the interview findings were used only to provide illustrative professional context for the survey results. Future research should include a larger and more diverse sample of veterinarians from different countries, practice settings, and career stages to allow a more robust qualitative or mixed-methods analysis of veterinary perspectives on euthanasia decision-making. The interviews provided further insight into the ethical complexity of euthanasia decision-making. Both veterinarians emphasized animal welfare as the primary ethical priority. However, notable differences emerged regarding how pet owner circumstances should influence clinical decisions. The senior veterinarian adopted a predominantly medical and diagnosis-based approach, whereas the early-career veterinarian incorporated broader social and emotional considerations. These differing perspectives may reflect generational, educational, cultural, or experiential influences, highlighting that ethical decision-making in veterinary medicine is not universally standardized. Similar ethical variation has been described previously among practicing veterinarians and veterinary students [13,31].

Interestingly, neither age nor gender was significantly associated with pet owner emotional burden in the present study. This suggests that emotional distress related to euthanasia may be more strongly influenced by relationship quality, prior experiences, and perceived professional support than by demographic characteristics alone. Future studies should therefore investigate additional psychosocial predictors, including attachment style, caregiving burden, previous bereavement experiences, and cultural attitudes toward animal death.

The findings of the study have practical implications for veterinary education and clinical practice. Structured communication training, routine implementation of quality-of-life assessment tools, and improved access to emotional support systems may facilitate more informed and ethically balanced euthanasia decision-making. In addition, the results support the integration of communication skills, ethical reasoning, and emotional resilience training into veterinary education and continuing professional development programs.

This study has several limitations. The recruitment strategy may have introduced self-selection bias. Because participants were recruited through social media and participation was voluntary, the sample may overrepresent pet owners who were more emotionally engaged, more affected by euthanasia experiences, or more interested in animal welfare and end-of-life decision-making. Therefore, the findings should not be interpreted as representative of all pet owners experiencing companion animal euthanasia, but rather as reflecting the experiences of a self-selected exploratory sample. The availability of the questionnaire only in English and German represents an additional limitation. Although the publicly accessible survey link allowed responses from several countries, the absence of further language versions may have limited participation from owners in non-English- and non-German-speaking contexts and may have influenced the geographical composition of the sample. Future studies should include validated translations in additional languages to allow more balanced cross-cultural comparisons. The survey relied on voluntary participation and included a predominantly younger and highly engaged pet owner population, with many respondents originating from Germany, which may limit generalizability. Species-specific and disease-specific variables were not differentiated, and the qualitative component involved only two veterinarians. Consequently, the findings should be interpreted as exploratory rather than universally generalizable. Despite these limitations, this study provides valuable insight into the emotional, ethical, communicative, and clinical dimensions of companion animal euthanasia. Future research should include larger multicenter populations, cross-cultural comparisons, species-specific analyses, and longitudinal investigations to better understand how communication, ethical conflict, and emotional support influence end-of-life decision-making over time. Several relevant dimensions of euthanasia decision-making could not be examined in detail within the scope of the present study. These included species-specific differences, diagnosis and disease stage, previous euthanasia experiences, owner attachment style, cultural or religious attitudes toward animal death, financial constraints in greater depth, availability of palliative or hospice care, and owners’ prior familiarity with quality-of-life tools. In relation to veterinarians, the interviews addressed emotional burden and coping, but the small number of interview participants did not allow a detailed analysis of professional well-being, compassion fatigue, burnout, moral distress, institutional support, workload, or differences between practice types. These elements were left out or only briefly explored because the primary focus of the study was the pet owner survey, while the veterinarian interviews served an exploratory and contextual role. Future studies should examine these factors using larger veterinarian samples and dedicated instruments for professional emotional burden and moral distress. These factors may substantially influence euthanasia decision-making and should be incorporated into future research.

## 5. Conclusions

Companion animal euthanasia represents a complex clinical, ethical, and emotional decision-making process that extends far beyond medical assessment alone. The present study suggests that pet owner decisions are strongly influenced by emotional attachment, personal observation, and the quality of veterinary communication, while structured quality-of-life assessment tools remain underutilized in routine practice. Furthermore, perceived emotional support from veterinarians was associated with lower pet owner distress and greater satisfaction during the euthanasia process, highlighting the central role of compassionate communication in end-of-life care.

These findings emphasize that effective euthanasia decision-making requires not only clinical expertise, but also ethical sensitivity, communication competence, and emotional support for both pet owners and veterinary professionals. Greater integration of structured quality-of-life assessment, communication training, and emotional resilience strategies into veterinary education and clinical practice may improve animal welfare, support informed decision-making, and promote the long-term well-being of both pet owners and veterinarians.

## Figures and Tables

**Table 1:** Demographic characteristics of survey respondents and country distribution.

Characteristics	Category	*n*	%
Total respondents		228	100
Country	Germany	157	68.9
	Hungary	27	11.8
	South Africa	15	6.6
	Other countries	29	12.7
Age group	18–24 years	68	29.8
	25–34 years	57	25.0
	35–44 years	25	11.0
	45–54 years	23	10.1
	55–64 years	38	16.6
	>65 years	17	7.5
Gender	Female	183	80.3
	Male	44	19.3
	Not reported	1	0.4

Note: Country names were harmonized before analysis. For example, “Deutschland”, “Germany”, “DE”, and regional entries referring to Germany were coded as Germany. “Other countries” included Austria, France, Ireland, India, Switzerland, the United Arab Emirates, Israel, Malta, the Netherlands, Norway, South Korea, Spain, the United Kingdom, and the United States.

**Table 2:** Statistical comparisons of pet owner experience variables.

Comparison	Outcome Variable	Group 1	Group 2	Test Statistic and Effect Size	*p*-Value	Holm-Adjusted *p*-Value
Emotional support vs. no emotional support	Emotional burden score	3.36 ± 1.33, *n* = 207	4.45 ± 0.82, *n* = 11	*t* = −4.14 Cohen’s d = −0.83	0.001	0.002
Emotional support vs. no emotional support	Communication satisfaction score	1.79 ± 1.26, *n* = 207	3.36 ± 1.50, *n* = 11	*t* = −3.41 Cohen’s d = −1.24	0.006	0.006
Informed vs. not informed before euthanasia	Communication satisfaction score	1.81 ± 1.29, *n* = 206	3.32 ± 1.32, *n* = 22	*t* = −5.09 Cohen’s d = −1.17	<0.001	<0.001
Age group	Emotional burden score	-	-	F(5, 222) = 1.17 η^2^ = 0.026	0.325	Not applied
Gender	Emotional burden score	Female: 3.45 ± 1.37, *n* = 183	Male: 3.34 ± 1.26, *n* = 44	*t* = 0.50 Cohen’s d = 0.08	0.619	Not applied

Note: Emotional burden was rated on five-point Likert-type scales, where higher scores indicated greater emotional burden. Communication satisfaction was rated on five-point Likert-type scales, where lower scores indicated greater satisfaction. Values are presented as mean ± standard deviation. For emotional support comparisons, only explicit “Yes” and “No” responses were included. Cohen’s d was calculated for two-group comparisons, and eta-squared was calculated for the one-way analysis of variance. Holm–Bonferroni correction was applied to the three planned owner-experience comparisons. Age- and gender-related analyses were exploratory demographic comparisons and are reported descriptively.

**Table 3:** Main themes identified in open-ended pet owner feedback, with frequencies and illustrative examples.

Theme	Frequency *n* (%)	Illustrative Example
Quality-of-life, animal welfare, and avoidance of suffering	31 (29.2%)	“If the quality of life of your pet is being compromised, I support it 100%.”
Clearer information, explanation, and transparency	28 (26.4%)	“More thorough explanation of euthanasia.”
No specific improvement suggested or positive experience reported	27 (25.5%)	“I felt well informed.”
Empathy, emotional support, and compassionate communication	14 (13.2%)	“Gentle conversation and tenderness play a huge role in this emotional decision.”
Calm/private setting and sufficient time for farewell	12 (11.3%)	“Sufficient time to consider and say goodbye.”
Possibility of home euthanasia or familiar environment	9 (8.5%)	“I would do the process at home next time.”
Post-euthanasia care, bereavement support, memorial gestures, or child support	5 (4.7%)	“It would be amazing to have post-euthanasia care.”

Note: Percentages were calculated based on all respondents who provided open-ended feedback (*n* = 106). Because responses could contain more than one theme, frequencies do not sum to 100%. Illustrative examples were anonymized and translated into English where necessary.

**Table 4:** Summary of key themes identified in the two semi-structured veterinarian interviews, with illustrative examples.

Theme	Senior Veterinarian	Early-Career Veterinarian	Illustrative Examples
Decision basis	Medical necessity, diagnosis	QoL, prognosis, family context	Poor prognosis, severe suffering, no treatment option
QoL assessment	Clinical judgment	Clinical judgment + owner input	Pain, mobility, appetite, behavior
Communication	Early, clear explanation	Individualized emotional support	Diagnostics, chronic cases, owner adaptation
Emotional burden	Long-term/tragic cases	Young animals, children, repeated loss	Medical limits, family distress
Ethical conflict	Refusal of unjustified euthanasia	Owner misunderstanding	No indication, poor prognosis
Training needs	Limited formal preparation	Need for soft skills	Communication, ethics, scenarios

Note: The qualitative component included only two veterinarians and was used to provide illustrative professional context rather than generalizable qualitative conclusions. Examples are based on anonymized interview-derived material.

## Data Availability

The anonymized quantitative dataset supporting the findings of this study is available from the corresponding author upon reasonable request. Full interview transcripts are not publicly available due to privacy and consent restrictions. The survey questionnaire and interview guide are provided as Supplementary Materials.

## Supplementary Materials

The following supporting information can be downloaded at: https://www.mdpi.com/article/10.3390/ani16111738/s1, Supplementary File S1: Online pet owner questionnaire; Supplementary File S2: Semi-structured interview guide for veterinarians.
