# Supplementary material for: Factors Influencing Decision-Making in Companion Animal Euthanasia: A Mixed-Methods Study of Pet Owners and Veterinarians
Source: Animals (Basel). 2026 Jun 5;16(11):1738. doi: 10.3390/ani16111738 (PMC13256030; doi:10.3390/ani16111738)
Supplement: Supplementary file 1 [file animals-16-01738-s001.zip › S1_Online_pet_owner_questionnaire.pdf]

Supplementary File S1

Online Pet Owner Questionnaire

Factors Influencing Decision-Making in Companion Animal Euthanasia: A Mixed-Methods Study of Pet Owners and Veterinarians

This supplementary file presents the online questionnaire used to collect pet owner and caregiver responses. The questionnaire was distributed online and was available in English and German. The English wording is provided below.

Target respondents: Adult pet owners or caregivers who had made or experienced a companion animal euthanasia decision.

Consent and anonymity: Participation was voluntary and anonymous. Completion and submission of the questionnaire were considered informed consent to participate.

Questionnaire items

No.	Question	Response format / options
1	How old are you?	Single-choice. Response options: 18-24; 25-34; 35-44; 45-54; 55-64; >65.
2	What is your gender?	Single-choice. Response options: Woman; Male; Prefer not to say.
3	In which country are you living?	Open-ended text response.
4	Have you ever made the decision for the euthanasia of a pet or experienced it?	Single-choice. Response options: Yes; No.
5	What was the reason for the euthanasia?	Multiple-choice with optional free-text response. Response options included: Illness; Age-related decline; Aggression/behavior; Other.
6	How did you assess the animal's quality of life before making the decision to euthanize?	Multiple-choice with optional free-text response. Response options included: By observing my pet's behavior and condition; On the advice of the veterinarian; With a quality-of-life scale; Other.
7	Were there any external factors that influenced the decision to euthanize your pet?	Multiple-choice with optional free-text response. Response options included: Financial reasons; Emotional connection; Advice from family/friends/other people; Risk to other people; No external factors; Other.
8	How did the decision to euthanize your pet affect you emotionally?	Five-point Likert-type scale. Higher scores indicated greater emotional burden.
9	Do you think that euthanasia is acceptable for animals?	Single-choice. Response options: Yes; No.

No.	Question	Response format / options
10	How satisfied were you with the veterinarian's communication regarding euthanasia?	Five-point Likert-type scale. Lower scores indicated greater satisfaction with communication.
11	Were you informed by the veterinarian in advance about the procedure of euthanasia?	Single-choice. Response options: Yes; No.
12	Did you feel supported by your veterinarian in the decision-making process?	Single-choice with optional free-text response. Response options included: Yes; No; Not applicable/Other.
13	What improvements and comments would you suggest regarding the euthanasia decision-making process?	Open-ended text response.

Notes on response coding

- Multiple-response items were separated into individual binary variables before analysis.
- For emotional burden, higher Likert scores indicated greater burden.
- For satisfaction with veterinary communication, lower Likert scores indicated greater satisfaction.
- For the comparison of perceived emotional support from the veterinarian, only explicit “Yes” and “No” responses were included in the corresponding statistical analysis.
