# Supplementary material for: Factors Influencing Decision-Making in Companion Animal Euthanasia: A Mixed-Methods Study of Pet Owners and Veterinarians
Source: Animals (Basel). 2026 Jun 5;16(11):1738. doi: 10.3390/ani16111738 (PMC13256030; doi:10.3390/ani16111738)
Supplement: Supplementary file 1 [file animals-16-01738-s001.zip › S2_Semi-structured interview guide for veterinarians.pdf]

Semi-structured interview guide for veterinarians

1. How many years have you been practicing as a veterinarian?
2. What is your area of specialization?
3. How often do you perform euthanasia?
4. What factors influence your decision to perform euthanasia?
5. What tools do you use to assess an animal's quality of life?
6. Do you think that these tools are sufficient to determine the right time for euthanasia? Are you satisfied with the methods you apply?
7. How confident do you feel in determining the right time for euthanasia?
8. What is the biggest challenge you see in deciding on and performing euthanasia?
9. How do you approach the topic of euthanasia with pet owners? What are the biggest challenges in recommending euthanasia?
10. Do you offer care after euthanasia for pet owners?
11. Do you find it difficult to bring up the topic of euthanasia with pet owners?
12. How emotionally challenging is performing euthanasia for you? Do you feel prepared to handle the emotional challenges associated with euthanasia?
13. Do you think there are sufficient guidelines in veterinary medicine for decision-making regarding euthanasia?
14. Have you ever dealt with pet owners who disagreed with your suggestion to perform euthanasia? How do you handle such situations?
15. Have you ever been in an ethical dilemma, for example when a pet owner insists on treatment although you believe euthanasia would be the better option? How did you handle the situation?
16. Do you think that your veterinary medicine studies prepared you well for performing euthanasia?
17. How could veterinary universities prepare students better for performing euthanasia?
18. Is there anything you personally wish for to make your work in this area easier?
